# Genomic Scan for Runs of Homozygosity and Identification of Candidate Genes Under Domestication in Fengjing Pigs

**DOI:** 10.3390/life15121823

**Published:** 2025-11-28

**Authors:** Ligang Ni, Hepan Gan, Zhanpeng Gu, Siyuan Li, Junsheng Zhang, Chunbao Zhou, Xiaoyan Wang, Pan Xu

**Affiliations:** 1 School of Animal Science and Technology, Jiangsu Agri-Animal Husbandry Vocational College, Taizhou 225300, China; niligang805@163.com (L.N.); napzjs@163.com (J.Z.); zhou_tz@163.com (C.Z.); 2 College of Animal Science and Technology, Yangzhou University, Yangzhou 225009, China; 18894834407@163.com (H.G.); 19852511773@163.com (Z.G.); 221902215@stu.yzu.edu.cn (S.L.)

**Keywords:** runs of homozygosity, genomic inbreeding, economic trait, Fengjing pig

## Abstract

Fengjing pigs are a Chinese native breed known for their high reproductive ability. Runs of homozygosity (ROHs) have emerged as an effective tool for evaluating inbreeding levels and identifying relevant genes in selection. However, the declining population of Fengjing pigs in recent years has raised concerns about inbreeding. Therefore, this study aimed to investigate the ROH patterns, estimate genomic inbreeding levels, and identify candidate genes associated with economic traits using whole-genome resequencing data from 105 Fengjing pigs. A total of 2448 ROHs were identified, with an average of 23.31 ROHs per individual and an average length of 9.50 Mb. The inbreeding coefficient, based on ROHs, was 0.098. Additionally, three genomic regions with a high frequency of ROHs were identified. These regions contained 64 unique genes, including 14 genes associated with important economic traits. Moreover, six overlapping quantitative trait loci (QTLs) and four candidate genes (*HSPG2*, *CDC42*, *EPHB2*, and *GRHL3*) were identified on Sus scrofa chromosome (SSC) 6. These QTLs are associated with birth traits (health and reproductive efficiency) and meat development traits (meat quality and growth). This study identified many candidate genes and QTLs that overlapped with ROHs and are associated with economically significant traits. These findings can be used in future breeding, conservation, and utilization of specific Chinese native pig breeds.

## 1. Introduction

Runs of homozygosity (ROHs) refer to continuous homozygous regions in the genome of diploid organisms, which are formed when offspring inherit the same haplotypes from both parents. The length and frequency of ROHs can reflect the demographic and genetic history of a livestock population [1]. Related studies have suggested that ROHs may originate from more closely related ancestors. The longer the ROH region, the higher the likelihood of recent inbreeding within a population. In contrast, shorter ROHs are derived from more distant ancestors, and their origins usually cannot be explained by existing genealogical records [2,3]. Many studies have also shown that different mating systems, selection intensities, effective population sizes, and population structures in a livestock population can lead to the formation of distinct ROH patterns in the genome [4,5,6]. Therefore, ROHs can be used to estimate the inbreeding levels of livestock populations, infer their inbreeding history, identify selected genes and deleterious mutations, assess genetic diversity and the conservation of genetic resources, and optimize animal breeding strategies.

In recent years, with the widespread application of single nucleotide polymorphism (SNP) chips and whole-genome sequencing technology, ROH based research on livestock genomics has increased significantly [7]. Genes associated with growth rate, reproductive efficiency, immune function, and environmental adaptability have been identified in specific ROHs in the genomes of cattle [8,9], pig [10,11], and sheep [12]. Therefore, ROH detection is becoming increasingly prevalent in livestock population research and serves as an effective approach for identifying genes associated with important economic traits in livestock.

The Fengjing pig is an ancient indigenous pig breed of China, native to the Taihu Lake Basin. The Fengjing pig is characterized by early sexual maturity, good meat quality, and strong environmental adaptability. Its most prominent feature is its excellent reproductive performance, and it is also one of the breeds with the highest fecundity in the world. Owing to these outstanding breed characteristics, Fengjing pigs have been commonly used in hybrid breeding programs and scientific research projects. Over the past few decades, the population of Fengjing pigs has been declining due to the large-scale import of lean-type pigs from western countries for commercial production. Currently, the population of Fengjing pigs is estimated to be around 400–500, with the majority being concentrated in three state-supported conservation farms. A reduction in population size elevates the likelihood of inbreeding, which may increase the frequency of homozygous deleterious alleles and consequently impair individual phenotypic performance. However, there is limited research on the ROH patterns and inbreeding coefficients in the Fengjing pig population. Therefore, this study used the GeneSeek GGP Porcine 50K SNP chip to genotype 105 Fengjing pig samples, with the objectives of analyzing the genome-wide ROH patterns and inbreeding levels of Fengjing pigs, identifying high-frequency ROH regions, and detecting candidate genes associated with porcine economic traits. This study aims to contribute to the enhanced protection of the genetic diversity of Fengjing pigs and provide valuable insights for their effective utilization.

## 2. Materials and Methods

### 2.1. Population and Genotyping

The entire population of Fengjing pigs from a conservation pig farm in Jiangsu Province was used in this study, consisting of 105 individuals (99 females and 6 males). Ear tissue samples were collected from each individual, stored in 75% ethanol at −20 °C for subsequent genomic DNA extraction. Genomic DNA was isolated using a commercial DNA extraction kit (Tiangen Biotech, Beijing, China) in strict accordance with the manufacturer’s recommended protocol. The quality of the extracted DNA was assessed using a NanoDrop 2000 spectrophotometer (Thermo Fischer Scientific, Wilmington, DE, USA). DNA samples meeting the following criteria were used: a purity ratio (A260/280) between 1.7 and 2.0, and a DNA concentration higher than 50 ng/μL. Then, the qualified DNA samples were genotyped using the GeneSeek GGP Porcine 50K SNP chip (Neogen Corporation, Lansing, MI, USA).

For genotypic data processing, individuals with a SNP missing rate higher than 5% were removed, and only autosomal SNP markers were used for further analysis. The quality of the dataset was evaluated using the PLINK software (version 1.90) [13], with the following criteria: (1) individual call rate higher than 90%; (2) minor allele frequency (MAF) higher than 0.01; (3) Hardy–Weinberg equilibrium (HWE) *p*-value higher than 1.00 × 10^−6^. The observed heterozygosity (Ho) and expected heterozygosity (He) were also calculated using the PLINK software (version 1.90) [14].

### 2.2. ROHs Identification and Inbreeding Coefficient Estimation

ROHs were identified on the autosomes of each individual using the *R* package detectRUNS (version 0.9.6) (https://cran.r-project.org/web/packages/detectRUNS/vignettes/detectRUNS.vignette.html (accessed on 10 July 2024)), with the following specific detection criteria: (1) Minimum ROH length higher than 1 Mb; (2) Minimum number of consecutive SNPs per ROH higher than 50; (3) Maximum gap between consecutive SNPs in a ROH less than 1 Mb; (4) Maximum allowed number of missing genotypes per ROH is 5, and maximum allowed number of heterozygous genotypes per ROH is 1; (5) Sliding window threshold set to 0.05; (6) Minimum SNP density set to 1 SNP/50 kb.

Based on the length of ROH regions, all identified ROHs were categorized into three groups: 1–5 Mb, 5–10 Mb, and >10 Mb. For the entire population, the number of ROHs in each length category was counted, and the distribution of ROHs across individual chromosomes was determined. Additionally, for each individual, the total number of ROHs and the total length of all ROH regions were calculated and summarized. The genomic inbreeding coefficient based on ROHs (F_ROH_) for each individual was calculated using the formula described in published literature [15]: F_ROH_ = ΣL_roh_/L_auto_. Here, ΣL_roh_ represents the total length of all ROH regions in an individual, and L_auto_ denotes the total length of the autosomal genome covered by SNPs, which was determined to be 2.45 Gb in this study.

### 2.3. Detection of Genomic Regions with a High Frequency of ROHs

To identify genomic regions with a high frequency of ROHs, the proportion of SNPs located within ROHs was calculated. This was achieved by counting the number of times each SNP was found in ROH regions across all individuals. Then, the top 0.1% of SNPs with the highest frequency of occurrence in ROHs was selected as the threshold for defining genomic regions with a high frequency of ROHs.

Based on the pig reference genome (Sscrofa11.1), the genes located in these high-frequency ROH regions were annotated using ANNOVAR software (version 20200608) [16]. Subsequently, these annotated genes were subjected to Gene Ontology (GO) functional enrichment analysis and Kyoto Encyclopaedia of Genes and Genomes (KEGG) enrichment pathway analysis. The results were visualized using the clusterProfiler *R* package (version 4.4.4), with a *p*-value < 0.05 considered to indicate significant enrichment. Furthermore, the specific genes with significant functional enrichments were compared against the pig quantitative trait loci (QTL) database (Pig QTLdb) to identify candidate functional genes.

## 3. Results

### 3.1. Genetic Diversity and Inbreeding Coefficient

**Figure 1:**
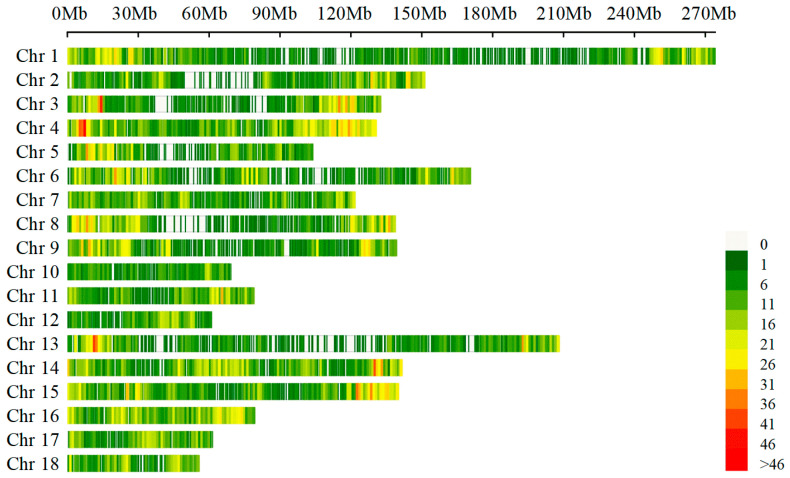
The SNPs density distribution on chromosomes. The x-axis represents the genomic position (Mb), and the y-axis represents the chromosomes. The density of SNPs within each genomic region is indicated by color.

After the analysis of the sequence datasets in this study, a total of 50,697 SNPs were identified. Following quality control, 21,442 informative SNPs were obtained. The distribution of these SNPs on chromosomes is illustrated in Figure 1. The number of SNPs varied across chromosomes, with the highest number (2047 SNPs) located on Sus scrofa chromosome (SSC) 1 and the lowest number (547 SNPs) located on SSC10.

The average observed heterozygosity (Ho) and expected heterozygosity (He) of the Fengjing pig population were calculated by genome-wide SNPs to be 0.330 ± 0.161 and 0.313 ± 0.148, respectively. To determine the inbreeding coefficient, F_ROH_ was used to assess the inbreeding status of the Fengjing pig population (Table 1). At the individual level, the average F_ROH_all_ value was 0.098, ranging from 0.008 to 0.200. Among the three inbreeding coefficients based on different ROH lengths, the F_ROH 1–5Mb_ displayed the lowest values. Meanwhile, F_ROH 1–5Mb_ had low correlations with F_ROH 5–10Mb_ and F_ROH>10Mb_ (Table S1).

### 3.2. ROHs Distribution Across the Genome

Among the 105 individuals, a total of 2448 ROHs were identified (Table S2). The average number of ROHs per individual was 23.31, with an average length of 9.50 Mb. The longest ROH, located on SSC1, spanned 68.95 Mb and contained 772 SNPs, while the shortest ROH, located on SSC13, measured 1.34 Mb and contained 54 SNPs. The ROHs were divided into three groups based on their length, as shown in Table 2. Of the total ROHs, 802 ROHs were shorter than 5 Mb, 869 ROHs ranged from 5 to 10 Mb, and 777 ROHs were longer than 10 Mb. The largest group was the ROHs with a length of 5 to 10 Mb, accounting for 35.50% of total ROHs.

**Figure 2:**
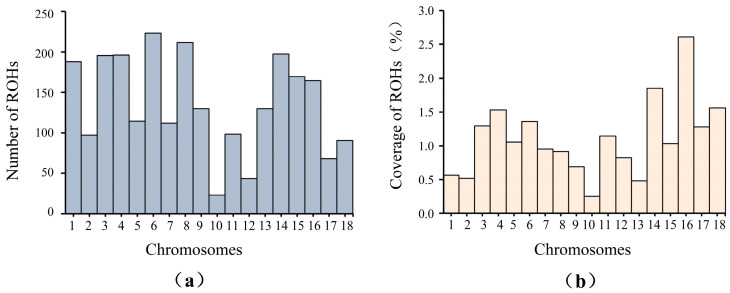
Distribution of ROHs on each chromosome. (**a**) Number of ROHs on each chromosome; (**b**) Coverage of ROHs on each chromosome.

The distribution of ROHs on each chromosome is shown in Figure 2. SSC10 had the lowest number of ROHs (23 ROHs), while SSC6 had the highest number of ROHs (233 ROHs) (Figure 2a). Furthermore, the coverage of ROHs for each chromosome was estimated. SSC10 displayed the lowest coverage of ROHs, while SSC16 displayed the highest coverage of ROHs (Figure 2b).

### 3.3. Genomic Regions with a High Frequency of ROHs

**Figure 3:**
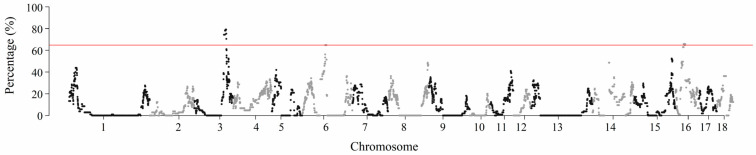
Manhattan plot of SNP frequency within ROHs across all individuals. The x-axis represents the genomic position on each chromosome; the y-axis represents the percentage of SNPs located within ROHs. The horizontal line indicates the threshold for the top 0.1% of SNPs with the highest frequency of occurrence in ROHs.

To identify genomic regions with a high frequency of ROHs, the frequency of SNPs in ROHs was calculated by tallying the number of times a SNP appeared in a specific ROH across all individuals. Based on this, the top 0.1% of SNPs with the highest frequency of occurrence in ROHs was selected (Table S3). The genomic positions of these SNPs on their respective chromosomes are presented in Figure 3. Three genomic regions with a high frequency of ROHs were identified (Table 3). These regions, located on SSC3, SSC6, and SSC16, contain 60, 32, and 33 SNPs, respectively, with lengths of 7.89 Mb, 3.46 Mb, and 5.32 Mb.

### 3.4. Candidate Gene Annotation and Enrichment Analysis

**Figure 4:**
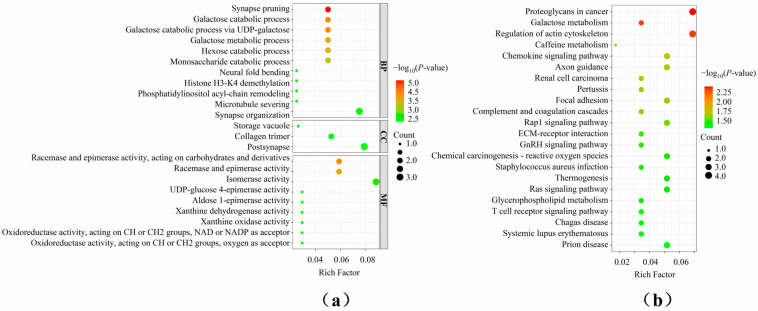
Functional enrichment analysis of candidate genes annotation in high-frequency ROH regions. (**a**) GO functional enrichment analysis of candidate genes; (**b**) KEGG enrichment pathway analysis of candidate genes.

To assess the selection signatures of genomic regions with a high frequency of ROHs, candidate genes located in these regions were annotated (Table S3). A total of 64 genes were identified, including 3 protein-coding genes and 5 long non-coding RNAs (lncRNAs). Functional enrichment analyses were then performed on these annotated genes (Figure 4). GO functional enrichment analysis revealed that these genes were involved in galactose catabolic process, galactose metabolic process, monosaccharide catabolic process, and others. Additionally, KEGG enrichment pathway analysis showed that these genes were significantly enriched in Galactose metabolism, Chemokine signaling pathway, Glycerophospholipid metabolism, T cell receptor signaling pathway, and others.

Furthermore, based on significant functional enrichments, genes associated with specific traits relevant to livestock breeding were screened. A total of 14 annotated genes were found to be associated with important economic traits (Table 3). Additionally, based on the pig QTLdb, eight QTLs associated with porcine economic traits were found to be overlapped with high-frequency regions (Table 4). Interestingly, six of these overlapping QTLs were located on SSC6, including QTL:21528 and QTL:21527 (both in *HSPG2* gene sequences), QTL:178770 (in *CDC42* gene sequences), QTL:179298 and QTL:179297 (both in *EPHB2* gene sequences), and QTL:284953 (in *GRHL3* gene sequences).

## 4. Discussion

The occurrence and distribution of ROHs in a population are influenced by selection, recombination, and population history [1,6]. ROHs are commonly used to assess the level of inbreeding across the entire genome in animal populations and to identify special genomic regions under selection [17,18]. In this study, we used the GeneSeek GGP Porcine 50K chip to investigate the occurrence and distribution of ROHs in the Fengjing pig population. Our goal was to analyze ROH patterns in Fengjing pigs and to detect candidate genes associated with economic traits.

### 4.1. Analysis of Genetic Diversity and Inbreeding Coefficient

Previous studies have suggested that the value of Ho and He are indicative of population genetic diversity [19]. In this study, we observed that Ho was slightly higher than He in the Fengjing pig population, and both values were close to 0.3. This may indicate higher genetic diversity in the Fengjing pig population. Moreover, genome-wide SNPs are widely used for assessing population inbreeding [20], and F_ROH_ mainly determined by the ratio of the total length of homozygous regions to the autosomal genome length [21]. In our study, the inbreeding coefficients were estimated based on ROHs. The results showed that the F_ROH_ value for the all ROH length category in the Fengjing pig population was relatively high, at 0.098. This result may be attributed to the small effective population size, as a reduced population size is known to promote increased levels of inbreeding [22]. However, the F_ROH_ value for the 0.5–1 Mb ROH length category was lower than those of the other length categories. This suggesting that a reduced effective population size in recent generations was the primary reason for the increase in inbreeding coefficient. When compared to commercial pig breeds, the F_ROH_all_ value of the Fengjing pigs was lower than that of the Large White pigs (0.140) [23] and Pietrain pigs (0.205) [24]. This phenomenon may indicate that the Fengjing pig population has not undergone intensive artificial selection, thereby retaining a certain level of genetic diversity.

### 4.2. Analysis of General Characteristics of ROHs

Many studies have demonstrated that ROHs may originate from more closely related ancestors, and the genomic distribution patterns of ROHs serve as an indicator of differences in breed management and formation [4,5]. The length of ROHs reflects the historical origins of breed populations: short ROHs are indicative of ancient inbreeding, while long ROHs suggest recent inbreeding [2,3]. In our study, the majority (approximately 68%) of ROHs detected in the Fengjing pig population were classified as short or medium length (<10 Mb). This finding is consistent with previous studies on pigs [17,23,24]. However, long ROHs (>10 Mb) still accounted for a relatively large proportion (31.74%) of the total ROHs, exceeding the corresponding proportions reported for Jinhua pigs (7.56%), Erhualian pigs (10.58%), and Meishan pigs (10.08%) [5,11]. This result suggests that the effective population size of the Fengjing pig population has been small in recent generations, resulting in a higher frequency of long ROHs. In fact, conservation efforts for Jinhua pigs, Meishan pigs, and Erhualian pigs have been more successful in recent generations, which is accompanied by their larger effective population sizes. Additionally, it is worth noting that population bottlenecks and genetic drift can also have an impact on the formation of ROHs [25].

### 4.3. Analysis of Genomic Regions with a High Frequency of ROHs and Candidate Genes

Previous studies have established consensuses regarding the role of ROHs in animal populations. Specifically, the distinct distribution patterns of ROHs occurred by artificial selection or domestication, and genomic regions with a high frequency of ROHs may indicate potential selection signatures for specific traits [4,6]. In this study, we detected three genomic regions with a high frequency of ROHs (The top 0.1% of SNPs with the frequency of occurrence in ROHs), and 64 candidate genes were identified in these regions. Functional enrichment analyses showed that these genes were enriched in the GO term “galactose metabolic process” and in KEGG pathways, including “Galactose metabolism, “Chemokine signaling pathway”, “Glycerophospholipid metabolism”, and “T cell receptor signaling pathway”. These results indicate that the functional genes detected in this study are closely related to the meat quality, growth, reproduction and health of pigs.

Based on significant functional enrichments, we identified several candidate genes in Fengjing pigs that may be associated with the traits of interest. Three candidate genes related to growth traits: *LTBP1*, *BIRC6*, and *ITGA2*. Among them, *LTBP1* is an important signaling molecule in the TGF-β pathway, involved in regulating the growth and physiological processes of skeletal muscle in mammalian bodies [26,27]. The *BIRC6* gene plays a role in cell apoptosis and cytoplasmic division and has been shown to affect animal fertility, average daily gain, and feed efficiency [28,29]. Four candidate genes related to reproduction traits: *BIRC6*, *KIF17*, *CDC42*, and *EPHB2*. Among them, *BIRC6* has been confirmed to be involved in the development of animal follicles and early embryonic development [30,31]. *KIF17* belongs to the kinesin-2 family and plays an indispensable role in mammalian spermatogenesis [32]. Previous studies have demonstrated that *KIF17* colocalizes with its cargo proteins in the manchette and the principal piece of the sperm tail and was implicated in spermatogenesis [33]. Four candidate genes related to meat traits: *LCLAT1*, *FGF10*, *HSPG2*, and *NNT*. Among them, *LCLAT1*, a microsomal enzyme involved in phospholipid remodeling, has been identified as a candidate gene associated with pork quality (Intramuscular fat content) [34]. *FGF10*, a member of the FGF family involved in various developmental processes, has been identified as a candidate gene associated with meat color traits in pigs [35]. Four candidate genes related to health traits: *MUL1*, *CCL28*, *C5orf34*, and *GRHL3*. Among them, *MUL1* was an immune-related protein gene that participates in regulating the body’s innate immune response to against viral infections by inhibiting the RIG-I-dependent antiviral response [36]. *CCL28* functions as an immune mucosal protein that binds to CCR3 receptors, driving lymphocyte migration, initiating immune response, and clearing pathogens [37]. These results indicate that genomic regions with a high frequency of ROHs in Fengjing pigs are associated with key selected production traits (growth, reproduction, meat quality, and health traits), these traits are directly shaped by long-term environmental conditions and artificial selection.

Finally, by searching the pig QTLdb, we have identified eight QTLs overlapping with high-frequency ROH regions that are associated with economically important traits in pigs. Interestingly, six of these QTLs were located on SSC6, residing within the *HSPG2*, *CDC42*, *EPHB2,* and *GRHL3* gene sequences, respectively. The *HSPG2* gene encodes a large proteoglycan that is a component of the extracellular matrix and has been identified as a candidate gene for fat deposition and growth traits, QTL (QTL:21528) in *HSPG2* gene sequence associated with growth (Days to 113 kg) were identified [38]. Moreover, Duroc pigs with the GG genotype (QTL:21527 in *HSPG2* gene sequence) grew more slowly and had higher marbling scores [38]. *CDC42* is expressed in the trophoblast cells of the placenta and participates in the migration of trophoblast cells [39], QTL (QTL:178770) in *CDC42* gene sequence associated with litter size have been reported [40,41]. *EPHB2* belongs to the Eph family of receptors, which plays an important role in pig embryo implantation and may affect reproductive efficiency [42], and two QTLs (QTL:179298; QTL:179297) in *EPHB2* gene sequence associated with litter weight were identified [43]. *GRHL3* belongs to the Grainyhead-like (GRHL) transcription factor family and plays a regulatory role in cell proliferation and apoptosis [44], QTL(QTL:284953) in *GRHL3* gene sequence showed a significant association with susceptibility to umbilical hernia in pigs [45]. These results reveal that the overlapping QTLs detected on SSC6 in the present study are strongly associated with birth traits (health and reproductive efficiency) and meat development traits (meat quality and growth). This finding also reflects the unique characteristics and historical selection effects of Chinese native pigs. Meanwhile, these QTLs are all located in the high-frequency ROH regions on SSC6. This indicates that the homozygous genomic region on SSC6 is a core region under selection in the Fengjing pig population and a key target for future pig breeding.

## 5. Conclusions

In this study, we investigated the ROH patterns and genomic inbreeding level of the Fengjing pig population using whole-genome resequencing data. A total of 2448 ROHs were identified. Through the analysis of these ROHs, we identified three genomic regions with a high frequency of ROHs. These regions contained 64 unique genes, including 14 genes associated with important economic traits. By searching the pig QTLdb, we identified eight QTLs that overlapped with the high-frequency ROH regions; six of these QTLs were located on SSC6, residing within the *HSPG2*, *CDC42*, *EPHB2*, and *GRHL3* gene sequences. These QTLs are associated with various traits such as growth, reproduction, meat quality, and health. Our findings enhance the understanding of genetic diversity, genome structure, and the genetic mechanisms underlying economically important traits in the Fengjing pig population.

## Figures and Tables

**Table 1:** The inbreeding coefficient of the Fengjing pig population.

Inbreeding Coefficient	Number of Animals	Mean	SD Standard Deviation	Minimum	Maximum
F_ROH_all_	105	0.098	0.043	0.008	0.200
F_ROH > 10 Mb_	105	0.061	0.026	0.005	0.154
F_ROH 5–10 Mb_	105	0.034	0.012	0.003	0.053
F_ROH 1–5 Mb_	105	0.012	0.006	0.002	0.024

**Table 2:** Statistics of ROH number and length based on ROH length.

ROH Length (Mb)	ROH Number	Percent (%)	Mean Length (Mb)	Standard Deviation
1–5	802	32.76	3.64	0.86
5–10	869	35.50	7.04	1.43
>10	777	31.74	18.29	9.41
Total	2448	100.00	9.50	8.19

**Table 3:** Statistics of the genomic regions with a high frequency of ROHs. A high-frequency ROH region was defined as a genomic segment containing SNPs ranked within the top 0.1% for frequency of occurrence in ROHs.

Chromosome	Position (Mb)	Length (Mb)	Number of SNPs	Candidate Gene Related to Economic Trait
3	101.15–109.04	7.89	60	*LTBP1*, *BIRC6*, *LCLAT1*
6	78.76–82.21	3.46	32	*MUL1*, *HSPG2*, *CDC42*, *EPHB2*, *GRHL3*, *KIF17*
16	27.61–32.93	5.32	33	*FGF10*, *CCL28*, *C5orf34*, *NNT*, *ITGA2*

**Table 4:** Statistics of overlapping QTLs associated with porcine economic traits based on the pig QTLdb.

Chromosome	Gene	Position (Mb)	QTL	Porcine Economic Trait
3	*LCLAT1*	108.69–108.88	QTL:22438; QTL:22439	Intramuscular fat content
6	*HSPG2*	79.85–79.96	QTL:21528; QTL:21527	Days to 113 kg; Marbling
6	*CDC42*	80.04–80.10	QTL:178770	litter size
6	*EPHB2*	80.65–80.84	QTL:179298; QTL:179297	Litter weight
6	*GRHL3*	82.01–82.05	QTL:284953	Umbilical hernia

## Data Availability

The original contributions presented in this study are included in the article and Supplementary Materials. Further inquiries can be directed to the corresponding authors.

## Supplementary Materials

The following supporting information can be downloaded at: https://www.mdpi.com/article/10.3390/life15121823/s1, Table S1: Pearson correlation coefficients between inbreeding coefficients; Table S2: Detection of ROHs in each genome; Table S3: The list of the top 0.1% of SNPs with the highest frequency of occurrence in ROHs.
