# Supplementary material for: Genomic Scan for Runs of Homozygosity and Identification of Candidate Genes Under Domestication in Fengjing Pigs
Source: Life (Basel). 2025 Nov 28;15(12):1823. doi: 10.3390/life15121823 (PMC12735110; doi:10.3390/life15121823)
Supplement: Supplementary file 1 [file life-15-01823-s001.zip › life-3975205-supplementary/Supplementary File-life-3975205/Table S1.docx]

**Table S1.** Pearson correlation coefficients between inbreeding coefficients

| Correlation | F_ROH_all_ | F_ROH_ >10Mb | F_ROH_ 5-10Mb | F_ROH_ 1-5Mb |
| --- | --- | --- | --- | --- |
| F_ROH_all_ | 1 |  |  |  |
| F_ROH >10Mb_ | 0.953^**^ | 1 |  |  |
| F_ROH 5-10Mb_ | 0.662^**^ | 0.392^**^ | 1 |  |
| F_ROH 1-5Mb_ | 0.181^*^ | -0.005^ns^ | 0.081^ns^ | 1 |

^∗∗^Significantly different *p* < 0.01, ^∗^Significantly different *p* < 0.05, ^ns^: not significant
